# 15‐Hydroperoxy‐PGE_2_: Intermediate in Mammalian and Algal Prostaglandin Biosynthesis

**DOI:** 10.1002/anie.201910461

**Published:** 2019-10-23

**Authors:** Hans Jagusch, Markus Werner, Oliver Werz, Georg Pohnert

**Affiliations:** ^1^ Institute for Inorganic and Analytical Chemistry, Department of Instrumental Analytics/Bioorganic Analytics Friedrich Schiller University Jena Lessingstraße 8 07743 Jena Germany; ^2^ Institute of Pharmacy Department of Pharmaceutical/Medicinal Chemistry Friedrich Schiller University Jena Philosophenweg 14 07743 Jena Germany

**Keywords:** biosynthesis, inflammation, natural products, prostaglandins, structure elucidation

## Abstract

Arachidonic‐acid‐derived prostaglandins (PGs), specifically PGE_2_, play a central role in inflammation and numerous immunological reactions. The enzymes of PGE_2_ biosynthesis are important pharmacological targets for anti‐inflammatory drugs. Besides mammals, certain edible marine algae possess a comprehensive repertoire of bioactive arachidonic‐acid‐derived oxylipins including PGs that may account for food poisoning. Described here is the analysis of PGE_2_ biosynthesis in the red macroalga Gracilaria vermiculophylla that led to the identification of 15‐hydroperoxy‐PGE_2_, a novel precursor of PGE_2_ and 15‐keto‐PGE_2_. Interestingly, this novel precursor is also produced in human macrophages where it represents a key metabolite in an alternative biosynthetic PGE_2_ pathway in addition to the well‐established arachidonic acid‐PGG_2_‐PGH_2_‐PGE_2_ route. This alternative pathway of mammalian PGE_2_ biosynthesis may open novel opportunities to intervene with inflammation‐related diseases.

## Graphical abstract

**Inflamed**: A new pathway to a long‐known lipid mediator, PGE_2_, was discovered in the red alga *Gracilaria vermiculophylla* (see Figure, top). Upon re‐investigation of the biosynthesis in macrophages, 15‐hydroperoxy‐PGE_2_ was also established as an intermediate in an alternative pathway in mammals.

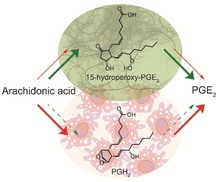

Arachidonic acid (AA) derived oxylipins are potent tissue hormones involved in numerous homeostatic biological functions, but also in the pathogenesis of a variety of disorders.^1^, ^2^ In mammals, these lipid mediators are generated in response to extracellular stimuli by cyclooxygenases (COXs) and/or lipoxygenases (LOXs).^3^ One class of these tissue hormones are the prostaglandins (PGs) that play a central role in regulating inflammation.^4^ Increase of PG levels during acute inflammation is part of the immune responses to injury and infections. Chronically elevated PG levels are connected to the pathogenesis of arthritis, cancer, atherosclerosis, stroke, and neurodegeneration.^2^, ^5^, ^6^ Nonsteroidal anti‐inflammatory drugs (NSAIDs) and coxibs (selective COX‐2 inhibitors) block the biosynthesis of PGs.^7^ PGE_2_ (**1**) is involved in diverse biological processes that include ovulation, bone metabolism, blood vessel tone, and pain.^8^ Its action is mediated by the G‐protein‐coupled receptors EP1‐4.^3^, ^9^ The well‐known AA‐PGG_2_‐PGH_2_‐PGE_2_ biosynthetic pathway involving COX‐1 and COX‐2 as key enzymes (red arrows Figure 1) is a widely accepted route to **1** and no alternative branches for the generation of **1** in mammals were considered until today.

**Figure 1:**
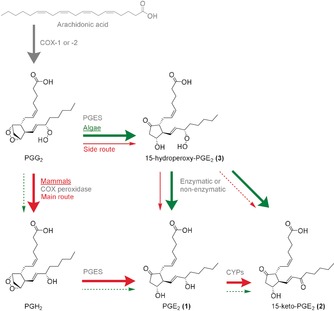
Biosynthetic pathways for PGE_2_ (**1**) and 15‐keto‐PGE_2_ (**2**) in mammals and algae. In the canonic pathway (thick red arrows), AA is converted by COX‐1 or COX‐2 into PGG_2_, which is subsequently reduced by the peroxidase activity of the COX to PGH_2_ and converted by PGE_2_ synthase (PGES) into **1**. In the novel pathway introduced here (thin red and thick green arrows), PGG_2_ is metabolized by a PGES‐like activity to the novel intermediate 15‐hydroperoxy‐PGE_2_ (**3**), which is reduced either enzymatically or non‐enzymatically to **1. 2** occurs as a metabolic by‐product. The contribution of both pathways to the formation of **1** differs between algae and mammals, probably due to different COX peroxidase activity.

Besides mammals, also marine algae generate AA‐derived oxylipins, including PGs that function primarily as defense molecules.^10^, ^11^ PGs derived from the red macroalga *Gracilaria vermiculophylla* are involved in the chemical defense against grazers of the alga and are therefore associated with its invasive spreading after introduction from the northwest Pacific.^12^, ^13^ The oxylipins are rapidly biosynthesized in response to wounding of the algal tissue. They can also be induced in intact algae upon reception of signals from pathogens and serve to increase resistance.^10^ Work on algal oxylipins has often stimulated novel concepts about oxylipin biosynthesis with implications for mammalian, plant, and algal pathways.^14^, ^15^ Edible red seaweeds of the genus *Gracilaria* contain high amounts of **1** related to numerous reported cases of food poisoning in East Asia upon ingestion of raw alga.^16^ The algal formation of **1** and metabolic intermediates are not fully elucidated yet. To explore the algal biosynthesis of **1**, we systematically mined *G. vermiculophylla* for AA‐derived oxylipins.

We collected fresh specimens of *G. vermiculophylla* from the Baltic Sea near Kieler Förde (Germany) for oxylipin extraction. To elucidate the complex oxylipin profile, we extracted *G. vermiculophylla* after mechanical wounding, a procedure that was previously shown to initiate the lipase‐ and LOX‐mediated formation of C20 oxylipins.^11^, ^13^ Briefly, the alga was frozen in liquid nitrogen and ground. After thawing and incubation at room temperature, the oxylipins were taken up in methanol. Solid‐phase extraction (SPE) provided samples for ultra‐high performance liquid chromatography coupled with mass spectrometry (UHPLC‐MS).^17^ We identified the common algal PGE_2_ (**1**) and 15‐keto PGE_2_ (**2**), as well as the novel putative oxylipin **3** (Figure 2).

**Figure 2:**
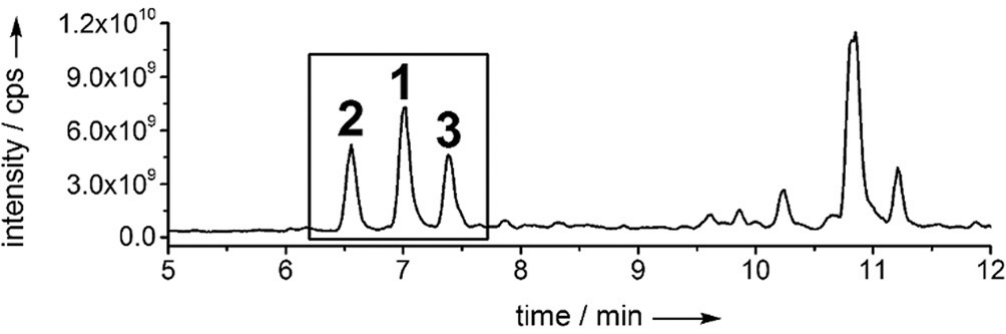
UHPLC‐MS profile of an extract from wounded *G. vermiculophylla*. The novel prostaglandin **3** elutes at 7.38 min next to **2** at 6.56 min and **1** at 7.02 min. Dihydroxy‐eicosatetraenoic acids (diHETEs) elute at 9.50–11.50 min. The oxylipin profile was recorded in negative ionization mode and the total ion count in full MS is plotted.

The high‐resolution mass of the pseudo molecular ion *m*/*z* 367.2116 [*M*−H]^−^ suggested an elemental composition of C_20_H_31_O_6_ (calculated mass 367.2120), consistent with a highly oxidized eicosanoid. For structure elucidation of **3**, we fractionated the algal extract by reversed‐phase high‐performance liquid chromatography (RP‐HPLC) and obtained 21.4 μg compound g^−1^ alga (fresh weight). The structure of **3** was assigned by 1D and 2D nuclear magnetic resonance (NMR) spectroscopy as well as by MS^2^ (see SI‐Figure 4 and SI‐Table 1 in the Supporting Information). A first comparison of NMR data suggested the molecule to be very similar to **1** with an intact 3‐hydroxy‐cyclopentanone core. The 5*Z*,13*E* double‐bond conformation was inferred by ^1^H,^1^H‐coupling. The UV spectrum with maximum absorption at 215 nm suggested a chromophore similar to that of **1** (see SI‐Figure 5).^18^ A hydroperoxy group at C15 was deduced from NMR and MS^2^ data: The ^13^C NMR signal of C15‐OOH (*δ*=85.4 ppm) was downfield shifted compared to C15‐OH of **1** at *δ*=70.7 ppm.^19^ The hydroperoxide **3** required lower energy for MS^2^ fragmentation compared to hydroxy functionalized **1**. The MS^2^ of **3** resembled that of **2**, which can be explained by an initial loss of water at C15 from **3** and further similar fragmentation (see SI‐Figure 4).

To determine the absolute configuration of **3**, we reduced the compound with NaBH_4_, resulting in **1** and PGF_2α/β_ (see SI‐Figure 2). Co‐injection with authentic standards confirmed the structures and further supported the hydroperoxy moiety in **3** (see SI‐Figure 3). The circular dichroism (CD) spectra of **1** and **3** isolated from *G. vermiculophylla* were similar and matched with the literature data for mammalian **1**, thus confirming an identical configuration of **3** and mammalian 15*S*‐configured **1** (see SI‐Figure 6).^20^ The novel oxylipin was thus elucidated as 15*S*‐hydroperoxy‐PGE_2_ (**3**). The structure and key NMR correlations are shown in Figure 3.

**Figure 3:**
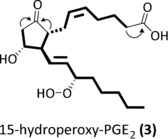
Structure of **3** with characteristic NMR correlations given as bold lines for ^1^H,^1^H‐COSY and as arrows for HMBC.

The hydroperoxide **3** was thermally unstable, forming **1** and **2** (confirmed with commercially available standards Figure 4; see SI‐Figures 1 and 3). The ratio of both metabolites generated upon heating of **3** to 37 °C for 24 hours was about 1:1, in accordance with a disproportion reaction. In wounded *G. vermiculophylla* this ratio was about 3:2, indicating an enzymatic contribution to their formation or metabolization.

**Figure 4:**
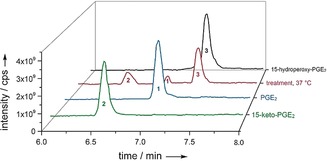
UHPLC‐MS profiles for **3** (black, red at 7.36 min) and its degradation products **2** and **1** (red at 6.5 min and 7 min) formed at 37 °C (for 24 h) in comparison to PG standards: **1** (blue) at 7 min; **2** (green) at 6.5 min. All profiles were measured in negative ionization mode and the total ion count in full MS is plotted. Note that the detector response for the ionization of **1** and **2** is different and that, after normalization with external standards, observed peak areas in the treatment at 37 °C correspond to equal molar amounts (see SI‐Figure 8 and SI‐Table 7).

In mammals, COX‐1 or COX‐2 catalyze the conversion of phospholipase‐released AA into PGG_2_. Subsequent transformation of the peroxide into PGH_2_ is mediated by the peroxidase activity of the COX enzymes.^21^ Subsequently, a PGES (three different isoenzymes are known) generates **1** from PGH_2_.^22^ Similar key enzymes were previously found in algae converting AA into **1**, however, isolation and elucidation of intermediates such as PGH_2_ was not reported.^23^, ^24^ The novel labile PG **3** can be explained by the action of a COX converting AA into PGG_2_, followed by a PGES‐like transformation of this intermediate. The abiotic conversion of **3** into **1** and **2** suggests it to be an alternative intermediate in the biosynthesis of these PGs in the alga. The product ratio of the algal PGs indicates a mixed mechanism involving disproportionation of **3** and its enzymatic transformation. An algal pathway that does not follow the established AA‐PGG_2_‐PGH_2_‐**1** route is supported by the fact that the COX of *G. vermiculophylla* shares just about 20 % of the amino‐acid sequence from the mammalian counterpart rendering the algal enzymes resistant to commonly available COX inhibitors.^23^ The peroxidase activity of the algal COX might be low, leading to the accumulation of PGG_2_. The algal PGES might then open the endoperoxide of PGG_2_ faster than the COX reduces the hydroperoxide preferably yielding **3**, thus privileging the novel route via **3** as compared to the well‐known (mammalian) pathway via PGH_2_.

Given the specificity of the abiotic transformation and the lability of **3**, we reasoned that this molecule might also represent an overlooked intermediate in mammalian PG biosynthesis. We, therefore, surveyed lipid mediators in human monocyte‐derived macrophages with an M1 phenotype that is known for substantial PG production during inflammation.^25^, ^26^ Stimulation of M1 with ionophore A23187 was used to induce PG biosynthesis.^27^ Lipid mediator extracts were screened for **3** using the UHPLC‐MS protocol described above. Indeed, **3** was detected (63 pg 2×10^6^ cells), besides **2** (109 pg 2×10^6^ cells^−1^) and **1** (5370 pg 2×10^6^ cells^−1^), in stimulated M1 macrophages (Figure 5). The amount of **3** increased upon A23187 stimulation, however it was still only detected in traces (ca. 1.2 %) compared to **1**. This observation would be in accordance with its role as an intermediate in PGE_2_ biosynthesis.

**Figure 5:**
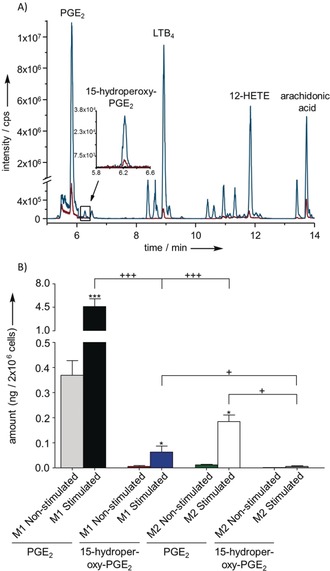
A) UHPLC‐MS profile of a lipid mediator extract from 2×10^6^ stimulated (blue) or non‐stimulated (red) human M1 macrophages measured in negative ionization mode, plotted as total ion count in full MS. The enhanced region shows the extracted ion chromatogram for **3**. The novel PG **3** elutes at 6.25 min. Further known lipid mediators were detected: **1** at 5.82 min, leukotriene B_4_ (LTB_4_) at 8.93 min, 12‐HETE at 11.85 min, AA at 13.73 min. B) Amounts of **1** (grey, black, green, white) and **3** (red, blue, orange, dark grey) produced in 2×10^6^ stimulated (*n*=4) or non‐stimulated (*n*=3) human M1 or M2 macrophages shown as means ± SEM. Cells were suspended in 1 mL PBS plus 1 mm CaCl_2_ and incubated for 10 min at 37 °C with 2.5 μm A23187 or vehicle (0.5 % methanol). Statistical evaluation: one way ANOVA with Tukey Post‐hoc test, *^/+^ *P*≤0.05; **^/++^ *P*≤0.01; ***^/+++^ *P*≤0.001, asterisks refer to comparison to non‐stimulated conditions.

Based on mechanistic considerations, **3** was postulated in the early 1970s as an intermediate in the sequence AA‐PGG_2_‐**3**‐**1** but has, to our knowledge, never been confirmed.^28^ Our finding now substantiates this pathway as an alternative route for the established sequence AA‐PGG_2_‐PGH_2_‐**1**.^29^ In comparison to M1 macrophages, we only found minor quantities of **3**, **2**, and **1** in M2 macrophages regardless of whether cells were stimulated or not. This data is consistent with the fact that M2 express only low levels of COX‐2 and PGES with moderate capacities to produce PGs (see SI‐Table 2).^30^

To confirm **3** as a direct precursor of **1**, we incubated either stimulated or nonstimulated M1 or M2 macrophages with **3** and analyzed the generated products by UHPLC‐MS. The amounts of **1** in both phenotypes were significantly elevated in samples amended with **3** (Figure 6; see SI‐Tables 2–4). The amount of **2** determined in the samples was lower compared to **2** arising from the disproportionation of **3** in the medium control. This finding is possibly due to metabolization of **2** by reductases to 13,14‐dihydro‐15‐keto‐PGE_2_ and eventually by β‐ and ω‐oxidation to PGE‐M (see SI‐Tables 3 and 4, and SI‐Figures 9 and 10).^31^ Although **3** accounts only for approximately 1.2 % in quantities compared to **1**, the contribution of this intermediate by the alternative biosynthetic branch to **1** is very likely higher because **3** is endogenously metabolized by human cells.

**Figure 6:**
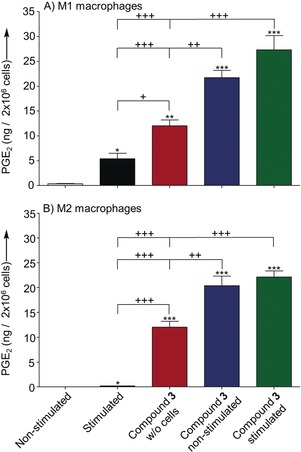
Production of **1** in 2×10^6^ stimulated or nonstimulated A) M1 or B) M2 macrophages, either treated with 100 nm 15‐hydroperoxy‐PGE_2_ (**3**; stimulated, non‐stimulated *n*=6, respectively) or vehicle (0.5 % methanol) (non‐stimulated *n*=3; stimulated *n*=4). Cells were suspended in 1 mL PBS plus 1 mm CaCl_2_ and preincubated with **3** or vehicle for 10 min at 37 °C. Cells were subsequently stimulated with 2.5 μm A23187 or vehicle (0.5 % methanol) and incubated for another 10 min at 37 °C. **3** was dissolved in 1 mL PBS plus 1 mm CaCl_2_ and incubated in absence of cells for 20 min at 37 °C as control (*n*=6) to determine formation of **1** resulting from degradation. All values are shown as means ± SEM. Statistical evaluation: one way ANOVA with Tukey Post‐hoc test, *^/+^ *P*≤0.05; **^/++^ *P*≤0.01; ***^/+++^ *P*≤0.001, asterisks refer to comparison to nonstimulated conditions.

In contrast to the transformation of **3** in the red macroalga *G. vermiculophylla*, where **1** and **2** were formed in a ratio of 3:2, M1 and M2 macrophages transformed **3** mainly into **1**, independently of A23187 stimulation. This data indicates a COX‐independent reducing activity in human cells. This process might be achieved either enzymatically by reductases or mediated by reductants such as glutathione (GSH). It was previously shown that the catalytic activity of COX is affected by cellular conditions, such as oxidative stress, altered substrate, and GSH or hydroperoxide concentrations (e.g., 15‐HPETE).^28^, ^32^, ^33^ We thus assume that the second pathway to **1** via **3** may be an adaption toward inflammation that affects these cellular conditions. For therapeutic approaches, selective inhibitors that address the COX peroxidase domain without affecting the cyclooxygenase domain may be developed. This approach might lead to inhibitors that reduce the carcinogenic effects caused by side‐products generated by COX peroxidase activity.^34^ These alternative inhibitors might also alleviate gastrointestinal and cardiovascular side effects observed for NSAIDs and coxibs.^35^ Regarding ingestion of raw alga, increased levels of leukotriene B_4_ and **1** are toxic for the gastrointestinal tract.^36^, ^37^ Both lipid mediators may be formed from labile precursors such as thermally unstable **3** or acid‐labile 5*R*,8*R*‐dihydroxy‐eicosatetraenoic acid.^17^

In conclusion, we reveal here an alternative biosynthetic pathway to PGE_2_ (**1**) in algae and in mammals. Upon cell activation, **1** is formed either by the canonical AA‐PGG_2_‐PGH_2_‐**1** route catalyzed by COX, COX peroxidase, and PGES or by the newly identified AA‐PGG_2_‐**3**‐**1** pathway mediated by COX, PGES, and hydroperoxide reduction. The contribution of each pathway to the production of **1** differs between algae and mammals. Whereas the first route via PGH_2_ dominates in mammals, algae preferentially rely on the second pathway via **3**, which represents a side route in human macrophages. These species‐dependent preferences are probably due to differences in the COX proteins affecting the peroxidase activity. Interestingly, **3** is reductively converted mainly into **1** in human macrophages but in macroalgae conversion leads into a mixture of **1** and **2**.

## Conflict of interest

The authors declare no conflict of interest.

## Supporting information

As a service to our authors and readers, this journal provides supporting information supplied by the authors. Such materials are peer reviewed and may be re‐organized for online delivery, but are not copy‐edited or typeset. Technical support issues arising from supporting information (other than missing files) should be addressed to the authors.

Supplementary
