# Supplementary material for: 15‐Hydroperoxy‐PGE2: Intermediate in Mammalian and Algal Prostaglandin Biosynthesis
Source: Angew Chem Int Ed Engl. 2019 Oct 23;58(49):17641–5. doi: 10.1002/anie.201910461 (PMC6899959; doi:10.1002/anie.201910461)
Supplement: Supplementary file 1 — Supplementary [file ANIE-58-17641-s001.pdf]

Angewandte Chemie
Eine Zeitschrift der Gesellschaft Deutscher Chemiker

Supporting Information

15-Hydroperoxy-PGE$_2$: Intermediate in Mammalian and Algal Prostaglandin Biosynthesis

Hans Jagusch, Markus Werner, Oliver Werz, and Georg Pohnert**

anie_201910461_sm_miscellaneous_information.pdf

Table of content:

1. Reagents 2
2. Isolation and structure elucidation of oxylipins 2
    - 2.1 Instrumentation 2
    - 2.2 Algae extraction 3
    - 2.3 Isolation 3
    - 2.4 Thermal degradation and reduction 3
    - 2.5 Profiling by LC-MS 3
    - 2.6 Analytical data for 15-hydroperoxy-PGE_2 6
3. Screening for oxylipins in human macrophages 8
    - 3.1 Isolation of human immune cells 8
    - 3.2 Differentiation and polarization 8
    - 3.3 Stimulation of macrophages for lipid mediator production 8
    - 3.4 Extraction 8
    - 3.5 LC-MS analysis and quantification 8
    - 3.6 SI-Tables and SI-Figures 9
4. Additional information 12
5. SI-References 14
6. NMR Spectra 14

1. Reagents

Solvents for chromatography and workup were obtained from VWR (France), Fisher Scientific (Sweden), and Carl Roth (Germany) in required quality grades. Deuterated solvents were purchased from Euriso-top (France). Deuterated and undeuterated lipid mediator standards were acquired from Cayman Chemical (United States). $NaBH_4$, ethylenediaminetetraacetate (EDTA), ionophore A23187, dextrane, lipopolysaccharide (LPS)–serotype 055B:B5, Roswell Park Memorial Institute (RPMI) 1640 medium, fetal bovine serum (FBS), a penicillin-streptomycin solution (P / S), and Ficoll-Histopaque 1077-1 were purchased from Sigma Aldrich (Germany). L-glutamine was obtained from Lonza (Switzerland), phosphate-buffered saline (PBS) from Serva (Germany), and granulocyte-macrophage colony-stimulating factor (GM-CSF), macrophage colony-stimulating factor (M-CSF), interleukin 4 (IL-4), and interferon gamma (INF-γ) from R&D Systems (United States).

2. Isolation and structure elucidation of oxylipins

2.1 Instrumentation

NMR

All 1D (^1H, ^{13}C) and 2D (HSQC, ^1H-^1H-COSY, HMBC) NMR spectra were recorded in deuterated solvents on an AVANCE III 600 MHz (equipped with an inverse coil) instrument (Bruker, Germany) at room temperature (22 °C). The chemical shifts are reported in parts per million (δ) relative to the resonance of the residual solvent signal (δ $(CD_3)_2SO$ = 2.50 for ^1H spectra and 39.51 for ^{13}C spectra). Coupling constants (J) are reported in Hertz (Hz). NMR spectra were evaluated using the TopSpin® software.

Analytical LC-MS

For UHPLC-MS, two instruments were used according to Jagusch *et al*. 2019.[1] Briefly, 1) A Dionex UltiMate® 3000 UHPLC (Thermo Fisher Scientific, Great Britain) coupled with a Q-Exactive® Plus Orbitrap mass spectrometer (Thermo Fisher Scientific) equipped with an electrospray ion source, an Acquity® UPLC BEH SHIELD RP_{18} column (2.1x100 mm, 1.7 µm, 130 Å, Waters, United States) and an UV/Vis-detector with a scan rate of 5 scans s^{-1} (wave lengths 180-800 nm) was used for untargeted oxylipin analysis. Following instrument settings were used: resolution: 280,000, sheath gas flow rate: 47, aux gas flow rate: 11, sweep gas flow rate: 2, capillary temperature: 254 °C, aux gas heater temperature: 408 °C, MS mode: negative, spray voltage: 3.3 kV, collision energy (CE): 10 or 30 eV, exclusion time: 2 s, MS / MS: MS^2-Top-Five: resolution: 17,000, MS^2 PRM: resolution: 17,000, target parent ion: 349 *m*/*z*, 351 *m*/*z*, 353 *m*/*z*, 367 *m*/*z*. The following elution gradient (1) was used: solvent A: 98 % H_2O + 2 % CH_3OH + 0.01 % CH_3COOH v/v/v, solvent B: 100 % CH_3OH + 0.01 % CH_3COOH v/v, column temperature: 50 °C, flow rate: 0.3 mL min^{-1}, gradient: 40 % to 85 % B in 12.5 min, 98 % B in 0.1 min, 98 % B for 2.9 min, 40 % B in 0.1 min, 40 % B for 0.9 min. For evaluation, the Xcalibur® software was used. 2) An Acquity® UPLC system (Waters) coupled with a QTRAP 5500 Mass Spectrometer (ABSciex, Germany) was used for targeted lipid mediator analysis (for analyte parameters see SI-Table 5).[2] The instrument was equipped with an electrospray ion source and a BEH® C18 column (2.1x100 mm, 1.7 µm, 130 Å, Acquity, United States). The machine was operated in scheduled or unscheduled multiple reaction monitoring (MRM) coupled with information-dependent acquisition (IDA) and an enhanced product ion scan (EPI). Following instrument settings were used: scheduled MRM window: 60 s, curtain gas: 35, collision gas: medium (MRM); high (EPI), MS mode: negative, ion spray voltage: -4000, temperature: 500 °C, ion source gas 1 and 2: 40, EPI exclusion time: 15 s, EPI threshold: 5000. The following elution gradient (2) was used: solvent A: 100 % H_2O + 0.01 % CH_3COOH v/v, solvent B: 100 % CH_3OH + 0.01 % CH_3COOH v/v, column temperature: 50 °C, flow rate: 0.3 mL min^{-1}, gradient: 42 % to 86 % B in 12.5 min, 98 % B for 3 min, 42 % B for 0.5 min. For evaluation, the Analyst® software was used.

Semi preparative LC

Semi-preparative HPLC fractionation was conducted on an Agilent system (HP 1100 series HPLC, United States) equipped with a LiChroCART® RP_{18e} column (10x250 mm, 5 µm, 100 Å, Merck Millipore, United States) coupled with an UV-detector (scan rate: 2 scans s^{-1}, wave lengths: 220-300 nm) according to Jagusch *et al*. 2019 and was slightly modified.[1] Following elution gradient was used for fractionation: solvent A: 98 % H_2O + 2 % CH_3OH + 0.01 % CH_3COOH v/v/v, solvent B: 100 % CH_3OH + 0.01 % CH_3COOH v/v, column temperature: 22 °C, flow rate: 4.0 mL min^{-1}, gradient (3) for prostaglandins: 65 % B for 16 min, 71.5 % B in 0.1 min, 71.5 % B for 39.9 min, 98 % B in 0.1 min, 98 % B for 4.9 min, 0 % B in 0.1 min, 0 % B for 4.9 min, 65 % B in 0.1 min, 65 % B for 5.9 min.

CD

CD measurements were performed on a J-810 CD spectrometer (Jasco, Germany). Following settings were applied: cell length: 0.1 cm, sensitivity: medium, wavelength range: 185-350 nm, scan speed: 100 nm s^{-1}. Methanol was used as a blank.

Drying and freeze-drying

Samples were dried on a TurboVap® (Biotage, Sweden) and an OIL MIST FILTER EMF3 high vacuum pump (Edwards High Vacuum Int., Great Britain). For freeze-drying an Alpha 1-2 LD Laborgefriertrockner (Martin Christ Gefriertrocknungsanlagen, Germany) was used.

Statistical and image processing software
For statistical evaluation and quantification the Excel and GraphPad Prism software was used. Chemical formulae were created with the ChemDraw software. As graphic processing programs GraphPad Prism and OriginPro were used.

2.2 Algae extraction

Algae collection and extraction was conducted according to according to Jagusch *et al*. 2019 and was slightly modified.[1] Briefly, field samples of the red alga *G. vermiculophylla* (Baltic Sea near Kieler Förde, Germany) were collected by hand in the intertidal region and frozen immediately in liquid nitrogen. Algae were characterized according to morphological features by the taxonomic expert Florian Weinberger (IFM Geomar, Kiel). Samples were stored at -80 °C until utilization. For extraction, the frozen tissue was taken out of the freezer (-80 °C) and ground in a mortar under liquid nitrogen. For analysis of intact tissue the cold sample was extracted. For samples of wounded algae, the homogenate was incubated for 20 min at 22 °C before transfer to 50 mL Falcon tubes. Ice-cold methanol was added in a ratio of 1:2 w/v.
The oxylipins were extracted by SPE according to a published protocol which was slightly modified.[3] The following steps were performed on ice. A sample Falcon tube was centrifuged on a Heraeus Multifuge® X3R (Thermo Fisher Scientific) for 5 min at 3,488 g. Subsequently, the supernatant was decanted into a fresh Falcon tube and diluted 1:4 v/v with water acidified with PBS-HCl (see SI-Table 8) and mixed thoroughly (final pH 3.5). The extract was loaded on a 10 g silica-bond C18 Vac cartridge (Sep-Pak®, 35 cc, 55-105 µm, 125 Å, WAT 043345, Waters) that was previously equilibrated with 20 mL methanol and 20 mL water. Next, the cartridge was washed with 20 mL water and 20 mL hexane. The oxylipins were eluted with 12 mL methyl formate into test tubes. The organic solvents were evaporated under a nitrogen stream. The residue was taken up in methanol-water (50:50 v/v for UHPLC-MS or 72:28 v/v for semi-preparative HPLC, maximum solubility: extract of 37 g alga mL^{-1}) and centrifuged at 3,488 g for 5 min (Heraeus Multifuge® X3R). The solution was transferred into a 2 mL Eppendorf tube and subsequently centrifuged twice at 21,130 g for 5 min in a Centrifuge 5424 R (Eppendorf, Germany). The purified extract was decanted into a new tube and stored in a freezer (-20 °C).

2.3 Isolation

The prostaglandins PGE_2 (**1**), 15-keto-PGE_2 (**2**), and 15-hydroperoxy-PGE_2 (**3**) were fractionated from the crude extract on a semi-preparative RP-HPLC using gradient 3. The isolates (**1**: 14.3-15.8 min; **2**: 12.2-13.8 min; **3**: 16.2-17.8 min) were either stored until drying at -20 °C (**1**, **2**) or at -80 °C due to thermal sensitivity (**3**). For long-term storage, the thermally instable prostaglandin **3** was freeze-dried, brought to dryness, dissolved in 1 mL methanol, transferred into a 4 mL vial, and stored under argon at -80 °C (**3**: 21.4 µg g^{-1} fresh weight alga). **1** and **2** were evaporated at 30 °C, brought to dryness, dissolved in 1 mL methanol, transferred into a 4 mL vial, and stored under argon at -20 °C (**1**: 33.1 µg g^{-1} fresh weight alga; **2**: 20.9 µg g^{-1} fresh weight alga).

2.4 Thermal degradation and reduction

Two different treatments with **3** were conducted: 1) The sample (stock in methanol, 69 mM) was diluted 1:1000 v/v in methanol-water (50:50 v/v). Four aliquots were prepared accordingly which were either incubated at -20 °C, 22 °C, 37 °C or 60 °C for 24 h. 2) 78.7 µL of the stock solution (in methanol, 69 mM) were diluted with 21.32 µL methanol v/v (final concentration 54.3 mM) and equimolar $NaBH_4$ was added thereafter. The sample was stirred at 0 °C for 5 min. The reaction was quenched with some droplets of water and the mixture was then diluted 1:500 v/v in methanol-water (50:50 v/v). All samples were analyzed by UHPLC-MS as indicated using gradient 1 (Q-Exactive Plus) with 3 µL injection volume.

2.5 Profiling by LC-MS

Nontargeted metabolomics experiments were performed with extracts (in methanol-water 50:50 v/v; 5 g algal extract mL^{-1}) from wounded or intact (data not shown) *G. vermiculophylla* that were analyzed *via* UHPLC-MS (Q-Exactive Plus) using gradient 1 with 1 µL injection volume.
Formed products of thermal degradation or reduction of **3** were analyzed *via* UHPLC-MS (Q-Exactive Plus) in comparison to various prostaglandin standards (**1**, **2**) and algal isolates (**1**) using gradient 1 (see SI-Figures 1-3). Therefore, isolated **3** (stock in methanol, 69 mM) and **1** (stock in methanol, 99.37 mM) were diluted 1:1000 v/v in methanol-water (50:50 v/v) and compared to the differently treated samples (-20 °C, 22 °C, 37 °C, 60 °C; $NaBH_4$), respectively. The following volumes were injected: 3 µL for all samples of **3** (69 µM), 1.8 µL of isolated **1** (99.4 µM), 3 µL of all prostaglandin standards (60 µM).

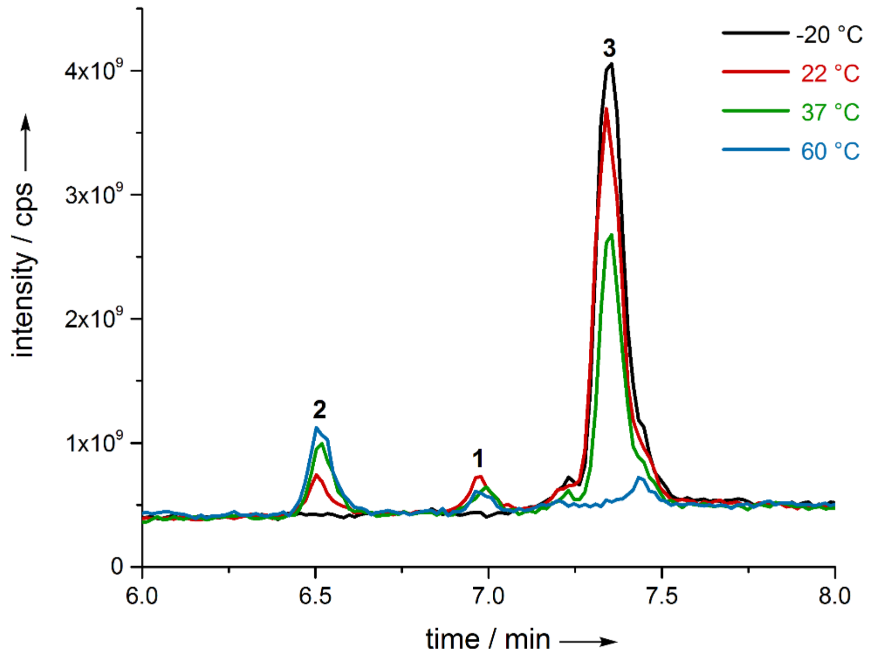

SI-Figure 1. UHPLC-MS profiles for 15-hydroperoxy-PGE_2 (**3**) (black, red, green at 7.36 min) and products 15-keto-PGE_2 (**2**) and PGE_2 (**1**) (red, green, blue at 6.5 min and 7 min, respectively) formed under thermally controlled conditions (black: -20 °C; red: 22 °C; green: 37 °C; blue:60 °C; 24 hours). All profiles were measured in negative ionization mode and the total ion count in full MS is plotted.

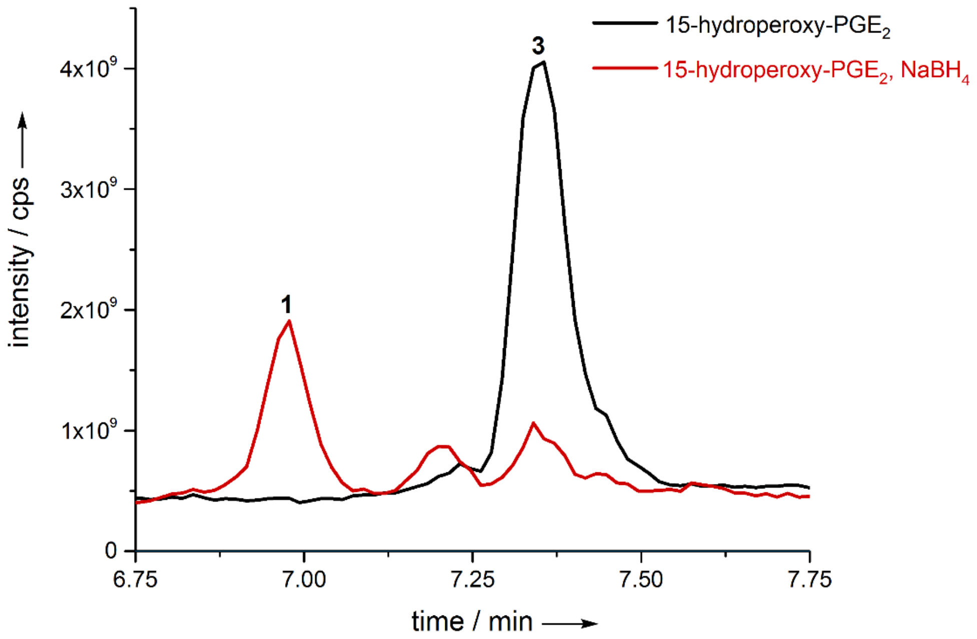

SI-Figure 2. UHPLC-MS profiles for **3** (black, red at 7.36 min) and products **1** and putative $PGF_{2\alpha/\beta}$ (red at 7 min and 7.2 min) formed upon treatment with $NaBH_4$. All profiles were measured in negative ionization mode and the total ion count in full MS is plotted.

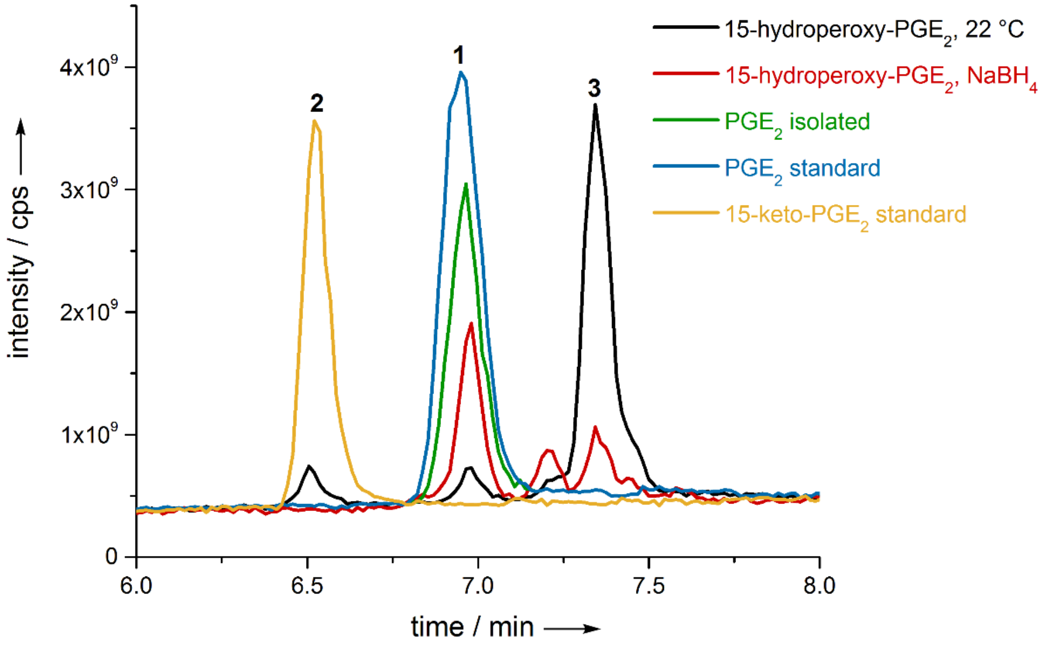

SI-Figure 3. UHPLC-MS profiles for **3** (black, red at 7.36 min) and products **2** (black at 6.5 min), **1** (black, red at 7 min), and putative $PGF_{2\alpha/\beta}$ (red at 7.2 min) formed under thermally controlled conditions (black: 22 °C, 24 hours) or upon treatment with $NaBH_4$ (red) in comparison to **1** isolated from alga (green) at 7 min and prostaglandin standards: **1** (blue) at 7 min; **2** (purple) at 6.5 min. All profiles were measured in negative ionization mode and the total ion count in full MS is plotted.

2.6 *Analytical data for 15-hydroperoxy-PGE$_2$*

The structure of **3** was assigned with MS^2 (Q-Exactive Plus, as indicated) and NMR (^{1}H, ^{13}C, HSQC, ^{1}H,^{1}H-COSY, HMBC) (see SI-Table 1). MS^2 fragmentation was conducted with 10 eV (see SI-Figure 4). The chromophore of **3** in comparison to **1** and **2** was confirmed by the UV spectrum showing a maximum absorption at 215 nm (see SI-Figure 5). The absolute configuration was deduced by LC and CD. Thermal degradation products of **3** co-eluted with the commercially available standards **1** and **2** (Q-Exactive Plus, as indicated). CD spectra of **3** and isolated **1** were in accordance with the commercially available standard **1** also excluding racemic mixtures (see SI-Figure 6).

SI-Table 1. ^{1}H, ^{13}C, and 2 D NMR (600 MHz, *DMSO-d$_6$*) for **3**:

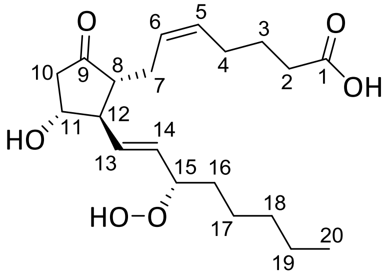

Number	δ_H (*J* in Hz)]		Multiplicity	δ_C	^{1}H,^{1}H-COSY	HMBC
1				174.9		
2	2.165		m	33.8	3	1, 3, 4
3	1.52	7.31	quin	24.94	2, 4	1, 2, 4, 5
4	1.98	7.31	q	26.69	3, 5	2, 3, 5, 6
5	5.36	7.31	dt	130.9	4, 6	3, 4, 6, 7
		10.80				
6	5.27	10.80	dt	127.2	5, 7	4, 5, 7, 8
		7.31				
7	2.23		m	24.95	6, 8	5, 6, 8, 9, 12
	2.18		m			
8	2.163		m	53.9	7, 12	6, 7, 9, 10, 11, 12, 13
9				215.4		
10	2.58	7.31	dd	47.2	10, 12	8, 9, 10, 12, 13
		18.31	dd			
	2.02	18.31				
		8.30				
11	3.99	8.30	q	71.3	8, 11, 13	7, 8, 9, 10, 11, 13, 14
12	2.31		m	53.0	12, 14	8, 11, 12, 14, 15
13	5.62	8.30	dd	134.6	13, 15	12, 13, 15, 16
		15.91				
14	5.43	15.91	dd	132.1	14, 16	13, 14, 16, 17
		7.51				
15	4.16	7.51	q	85.4	15, 17	14, 15, 17, 18
16	1.61		m	32.8	16, 18	15, 16, 18, 19
	1.37		m			
17	1.266		m	24.97	17, 19	16, 17, 19, 20
18	1.255		m	31.7	18, 20	17, 18, 20
19	1.261		m	22.6	19	18, 19
20	0.86	7.10	t	14.4	3	1, 3, 4

HRMS (ESI-) calculated for $[C_{20}H_{31}O_6\text{-}H]^-$ 367.2120, found 367.2116

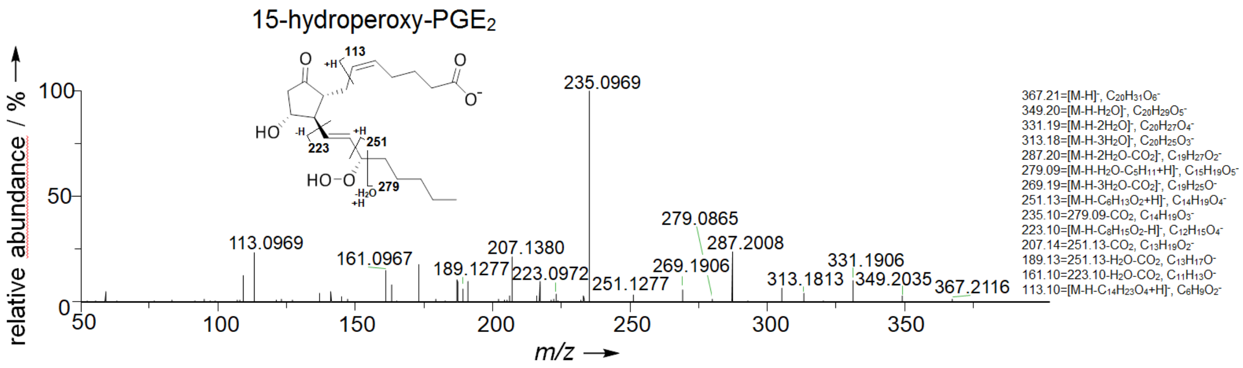

SI-Figure 4. Full MS^2 spectrum of **3**.

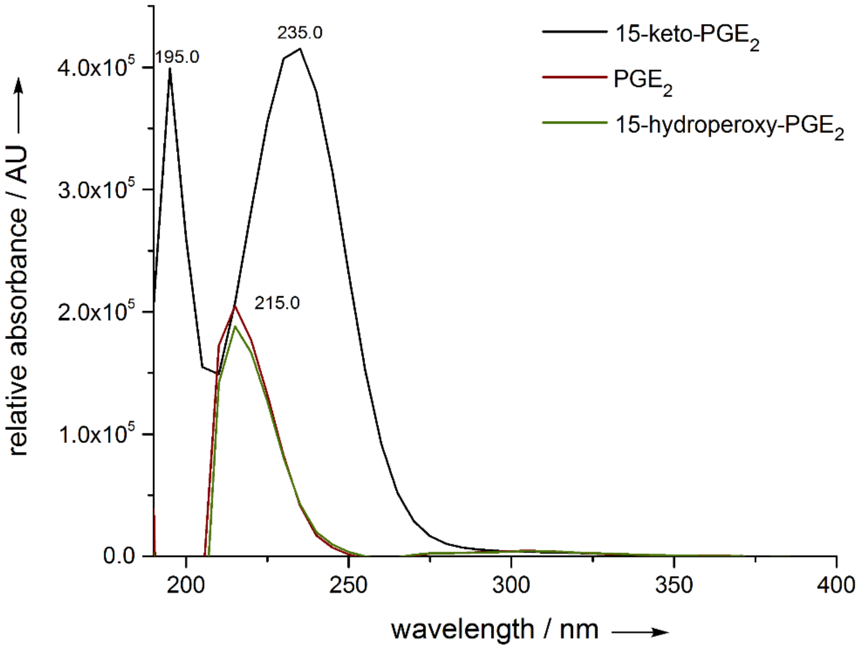

SI-Figure 5. UV spectrum for **1**, **2**, and **3**.

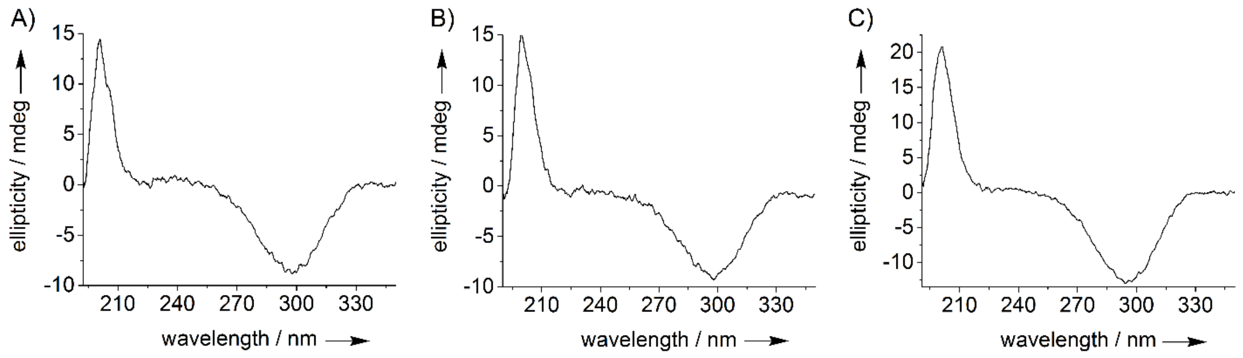

SI-Figure 6. CD spectra for A) **3**, B) isolated **1**, and C) standard **1**; 0.5 mL sample in methanol (final concentration 1 mM).

3. Screening for oxylipins in human macrophages

3.1 Isolation of human immune cells

Human peripheral blood mononuclear cells (PBMC) were collected from freshly withdrawn peripheral blood of healthy adult human donors, obtained from the Institute of Transfusion Medicine at the University Hospital Jena (Germany). The experimental protocol was approved by the local ethical committee at the University Hospital Jena. All methods were conducted in accordance with the relevant guidelines and regulations. Informed consent was given from all donors. PBMC were isolated as described.[2] Briefly, leukocyte concentrates were obtained from heparinized blood preparations by centrifugation (4,000 g, 20 min, 20 °C) on a Heraeus Multifuge® X3R (Thermo Fisher Scientific). Subsequently, leukocyte concentrates were subjected to dextran sedimentation and centrifuged (872 g, 4 °C, 10 min) on LymphoPrep. The PBMC fractions were combined and suspended in ice-cold PBS containing 0.1 % glucose (PG buffer) and counted on a Vi-CELL® XR (Beckman Coulter, United States).

3.2 Differentiation and polarization

For differentiation and polarization toward human M1 or M2 macrophages, published criteria were used.[3] Briefly, isolated PBMC were diluted and resuspended in PBS plus 1 mM $CaCl_2$ / $MgCl_2$ to 180x10^6 cells 15 mL^{-1} and incubated for 1 h (37 °C, 5 % CO_2) in a 175 cm flask. The medium was removed and 15 mL RPMI 1640 medium supplemented with 10 % FBS, penicillin-streptomycin (RPMIFP), and 5 mM L-glutamine were added to remove nonadherent lymphocytes. Monocytes were subsequently differentiated either to M0 macrophages with GM-CSF or M-CSF (20 ng mL^{-1}, respectively) for six days. Medium and differentiation agents were replaced after three days. After six days, the medium was removed and remaining cells were washed with 10 mL PBS. Adherent macrophages were detached using 10 mL PBS plus 5 mM EDTA and incubated for 30 min (37 °C, 5 % CO_2). The flask was shaken and cells were scraped into a 50 mL Falcon tube and spun down at 1200 g for 5 min at 4 °C. The pellet was taken up in 3 mL RPMIFP and macrophages were resuspended to obtain 2x10^6 cells 3 mL^{-1} per well in 6-well plates which were then incubated for 2 h (37 °C, 5 % CO_2). Polarization toward M1 or M2 macrophages was achieved applying 20 ng mL^{-1} INF-γ plus 100 ng mL^{-1} LPS or 20 ng mL^{-1} IL-4 followed by incubation for 48 h (37 °C, 5 % CO_2).

3.3 Stimulation of macrophages for lipid mediator production

Macrophages were stimulated to initiate lipid mediator production. Therefore, a well-established protocol was used and slightly modified.[2] Experiments were implemented with simplicates of a maximum of six different human donors. Briefly, RPMIFP was removed and 1 mL PBS plus 1 mM $CaCl_2$ was added per well and incubated for 3 min. Two approaches were applied for M1 and M2 macrophages: 1) general analysis of lipid mediator profiles (stimulated n=4, nonstimulated n=3), and 2) modification of the lipid mediator spectrum (n=6) using **3** (final concentration 100 nM) or methanol (0.5 %, vehicle) by preincubation for 10 min. The cells were then stimulated with ionophore A23187 (final concentration 2.5 µM) or methanol (0.5 %) for another 10 min. **3** was resolved in 1 mL PBS plus 1 mM $CaCl_2$ in the absence of cells (control) and incubated for 20 min (n=6). Lipid mediator biosynthesis was stopped with 2 mL ice cold methanol containing 10 µL deuterated standard mix (see SI-Table 6) for UHPLC-MS quantification.

3.4 Extraction

Solid phase extraction (SPE) of macrophage samples was conducted as published,[3] with slight modifications. The 3 mL incubation mixture (see above) was brought to -20 °C for 30 min to precipitate proteins. The sample was then centrifuged on a Heraeus Multifuge® X3R (Thermo Fisher Scientific) for 10 min at 1,200 g. Subsequently, the supernatant was decanted into a fresh Falcon tube and diluted 1:4 v/v with water acidified with PBS-HCl (see SI-Table 8) and mixed thoroughly (final pH 3.5). The extract was loaded on a 500 mg silica-bond C18 Vac cartridge (Sep-Pak®, 6 cc, 55-105 µm, 125 Å, WAT 043395, Waters) that was equilibrated with 6 mL methanol and 2 mL water. Next, the cartridge was washed with 6 mL water and 6 mL hexane. The lipid mediators were eluted with 6 mL methyl formate. The organic solvents were evaporated under a nitrogen stream. The residue was taken up in 200 µL methanol-water (50:50 v/v for UHPLC-MS) and centrifuged at 3,488 g for 5 min (Heraeus Multifuge® X3R). The solution was transferred into a 2 mL Eppendorf tube and subsequently centrifuged twice at 21,130 g for 5 min in a Centrifuge 5424 R (Eppendorf). The purified extract was decanted into a new tube and stored in a freezer (-20 °C).

3.5 LC-MS analysis and quantification

The lipid mediators produced by M1 or M2 macrophages were analyzed *via* UHPLC-MS (QTRAP) according to the literature.[2,4] A selected set of external standards (see SI-Table 7) was used. Standard curves for **3** and **2** were recorded separately (see SI-Figures 7 and 8). Six-point calibrations (0 nM, 5 nM, 25 nM, 50 nM, 100 nM, and 200 nM, n=3, respectively) with isolated **3** and commercially available **2** in methanol-water (50:50 v/v) were applied. Calculated values were normalized to the deuterated internal standard mix (see SI-Table 6). All samples were investigated using gradient 2 with 10 µL injection volume. Change in PG amounts upon treatment of cells with **3** were calculated as follows: the total amount of **1** or **2** in the respective cell type was subtracted by the amounts formed endogenously and by the amounts generated in the equally handled compound control. For statistical evaluation, amounts of PGs produced by differently treated M1 or M2 macrophages are shown as means ± SEM and a one way ANOVA with Tukey Post-hoc test was performed, * $P \le 0.05$; ** $P \le 0.01$, *** $P \le 0.001$.

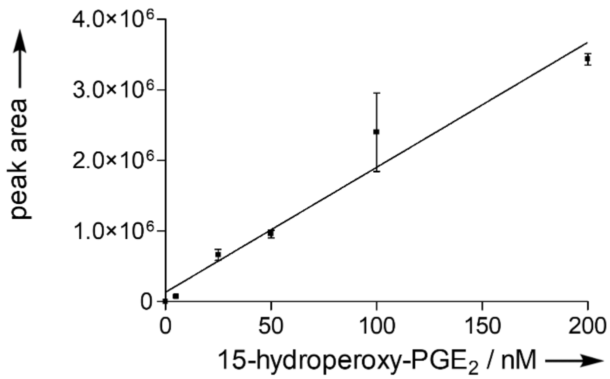

SI-Figure 7. Standard curve for quantification of isolated **3**. Calibration curve: y = 17677x + 7129000; R^2 0.9004.

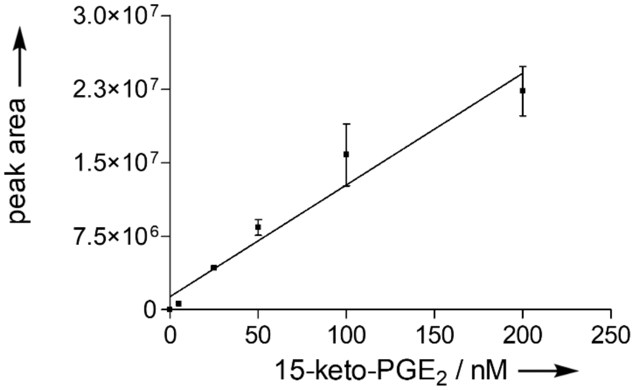

SI-Figure 8. Standard curve for quantification of commercially available **2**. Calibration curve: y = 114090x + 1329000; R^2=0.8788.

3.6 SI-Tables and SI-Figures

SI-Table 2. Formation of E_2 prostaglandins in M1 or M2 macrophages.

**[pg / 2x10⁶ cells]**	**M1 Stimulated**		**M1 Nonstimulated**		**M2 Stimulated**		**M2 Nonstimulated**	
	**Mean**	**± SE**	**Mean**	**± SE**	**Mean**	**± SE**	**Mean**	**± SE**
15-keto-PGE_2	108.8	43.0	5.2	0.9	21.4	6.0	1.2	0.4
15-hydroperoxy-PGE_2	62.8	24.1	7.9	3.1	5.7	2.8	0.9	0.6
PGE_2	5369.0	1114.4	385.0	77.7	184.0	26.4	12.5	4.1
Ratio 15-hydroperoxy-PGE_2 / PGE_2	1.2:99.8		2.1:97.9		3.0:97.1		6.7:92.3	
n=	4		3		4		3	

SI-Table 3. Formation of E_2 prostaglandins in M1 or M2 macrophages upon treatment with **3**.

[pg / 2x10^6 cells]	M1 Stimulated + 100 nM 15-hydroperoxy-PGE$_2$		M1 Nonstimulated + 100 nM 15-hydroperoxy-PGE$_2$		M2 Stimulated + 100 nM 15-hydroperoxy-PGE$_2$		M2 Nonstimulated + 100 nM 15-hydroperoxy-PGE$_2$		100 nM 15-hydroperoxy-PGE$_2$	
	**Mean**	**± SE**	**Mean**	**± SE**	**Mean**	**± SE**	**Mean**	**± SE**	**Mean**	**± SE**
15-keto-PGE$_2$	12172.9	3102.7	12903.1	1921.8	11532.9	1108.3	11536.9	2018.1	14280.7	2140.3
PGE$_2$	27296.9	2363.1	21755.1	1146.4	22106.4	1213.4	20380.1	1918.4	12039.9	1167.6
n=	6		6		6		6		6	

SI-Table 4. Additionally formed E_2 prostaglandins in M1 or M2 macrophages upon treatment with 15-hydroperoxy-PGE$_2$ **3**.

[pg / 2x10^6 cells]	M1 Stimulated + 100 nM 15-hydroperoxy-PGE$_2$		M1 Nonstimulated + 100 nM 15-hydroperoxy-PGE$_2$		M2 Stimulated + 100 nM 15-hydroperoxy-PGE$_2$		M2 Nonstimulated + 100 nM 15-hydroperoxy-PGE$_2$	
	**Mean**	**± SE**	**Mean**	**± SE**	**Mean**	**± SE**	**Mean**	**± SE**
15-keto-PGE$_2$	-2216.6	2688.9	-1378.8	2133.8	-2769.2	1557.2	-2744.57	1310.6
PGE$_2$	9888.1	2894.5	9346.8	1404.1	9882.4	1213.4	8328.3	1918.4
n=	6		6		6		6	

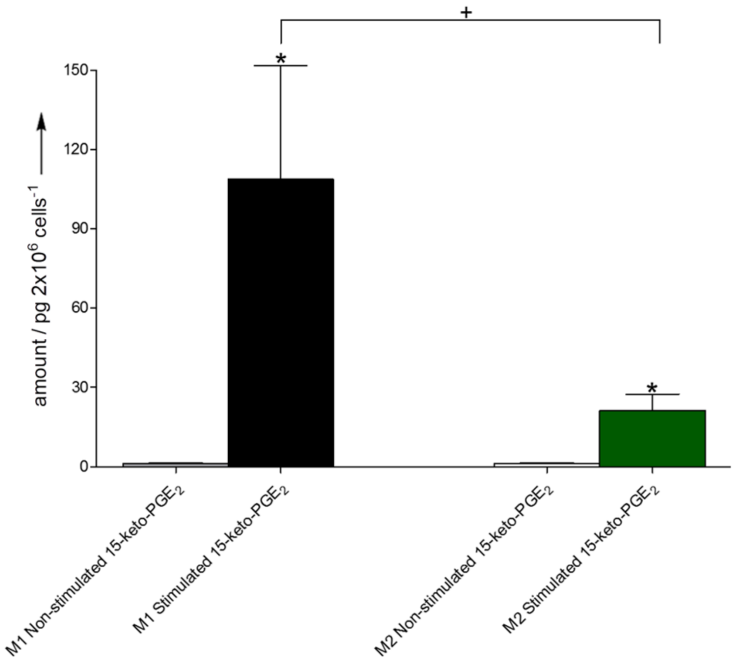

SI-Figure 9. Amounts of **2** produced in 2x10^6 stimulated (n=4) or nonstimulated (n=3) human M1 or M2 macrophages shown as means ± SEM. Statistical evaluation: one way ANOVA with Tukey Post-hoc test, $^{*/+}$ P≤0.05; $^{**/++}$ P≤0.01; $^{***/+++}$ P≤0.001. Cells were suspended in 1 mL PBS plus 1 mM $CaCl_2$ and incubated for 10 min at 37 °C with 2.5 µM A23187 or vehicle (0.5 % methanol).

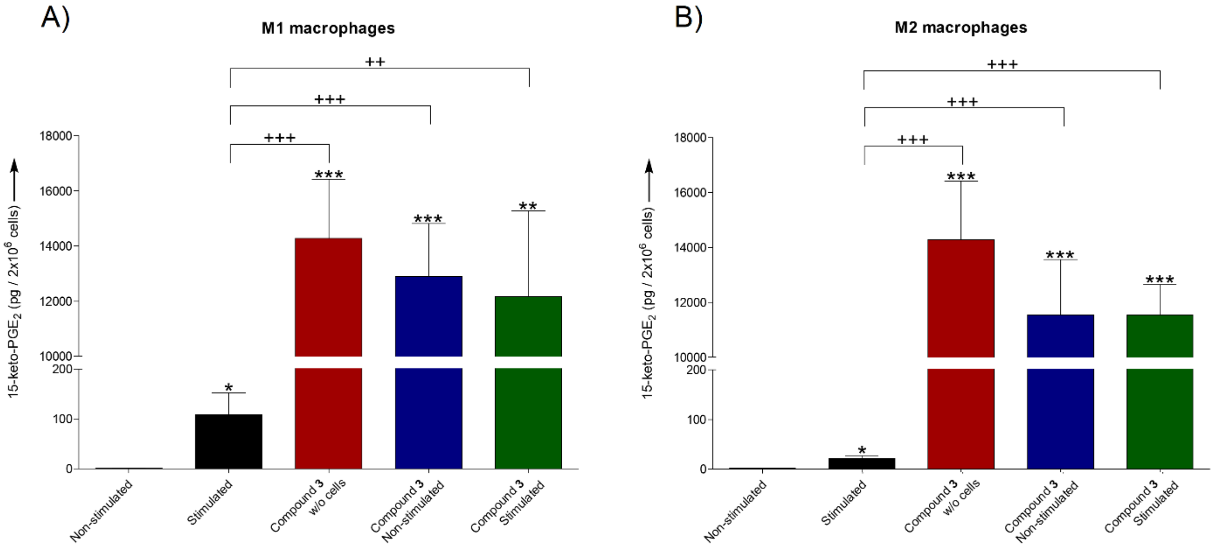

SI-Figure 10. Production of **2** in 2x10^6 stimulated or nonstimulated A) M1 or B) M2 macrophages, either treated with 100 nM **3** (stimulated, nonstimulated n=6, respectively) or vehicle (0.5 % methanol) (nonstimulated n=3; stimulated n=4). Cells were suspended in 1 mL PBS plus 1 mM $CaCl_2$ and preincubated with **3** or vehicle for 10 min at 37 °C, and subsequently stimulated with 2.5 µM A23187 or vehicle (0.5 % methanol) and incubated for another 10 min at 37 °C. **3** was dissolved in 1 mL PBS plus 1 mM $CaCl_2$ and incubated in absence of cells for 20 min at 37 °C as control (n=6) to determine formation of **2** due to degradation. All values are shown as means ± SEM. Statistical evaluation: one way ANOVA with Tukey Post-hoc test, $^{*/+}$ $P \leq 0.05$; $^{**/++}$ $P \leq 0.01$; $^{***/+++}$ $P \leq 0.001$.

4. Additional information

SI-Table 5. Analyte parameters for QTRAP measurements.

Q1	Q3	Retention Time [min]	Lipid Mediator	DP	EP	CE	CXP
327.3	116.1	12.0	d_8-5*S*-HETE	-80	-10	-17	-10
339.3	197.2	9.1	d_4-LTB_4	-80	-10	-22	-13
355.3	193.2	6.0	d_4-PGE_2	-80	-10	-25	-16
356.3	115.2	6.7	d_5-LXA_4	-80	-10	-19	-14
380.3	141.2	6.3	d_5-RvD_2	-80	-10	-23	-14
311.3	267.1	13.7	d_8-AA	-100	-10	-16	-18
375.2	121.1	6.7	RvD_1	-80	-10	-40	-13
375.2	215.1	6.7	RvD_1 2	-80	-10	-26	-13
375.2	141.1	6.3	RvD_2	-80	-10	-21	-13
375.2	175.1	6.3	RvD_2 2	-80	-10	-30	-13
375.2	147.1	6.3	RvD_3	-80	-10	-25	-13
375.2	181.1	6.3	RvD_3 3	-80	-10	-22	-20
375.2	255.1	7.7	RvD_4	-80	-10	-25	-18
375.2	101.1	7.7	RvD_4 2	-80	-10	-22	-10
349.2	195.1	4.2	RvE_1	-80	-10	-22	-12
349.2	161.1	4.2	RvE_1 2	-80	-10	-25	-13
351.2	115.1	6.7	LXA_4	-80	-10	-20	-13
351.2	235.1	6.7	LXA_4 2	-80	-10	-20	-13
349.2	115.1	5.7	LXA_5	-80	-10	-20	-13
349.2	215.1	5.7	LXA_5 2	-80	-10	-27	-13
351.2	221.1	6.2	LXB_4	-80	-10	-20	-13
351.2	251.1	6.2	LXB_4 2	-80	-10	-20	-13
349.2	221.1	5.3	LXB_5	-80	-10	-20	-13
349.2	251.1	5.3	LXB_5 2	-80	-10	-20	-13
351.3	189.1	6.0	PGE_2	-80	-10	-25	-14
351.2	271.1	6.0	PGE_2 EicoPAF	-120	-10	-20	-13
351.3	233.1	6.2	PGD_2	-80	-10	-16	-15
353.3	193.1	6.4	$PGF_{2\alpha}$	-80	-10	-34	-11
369.3	169.1	5.6	TXB_2	-80	-10	-22	-15
353.2	317.4	6.4	PGE_1 / PGD_1 I	-90	-10	-18	-15
353.2	273.4	6.4	PGE_1 / PGD_1 III	-90	-10	-28	-14
355.2	293.1	6.7	$PGF_{1\alpha}$	-75	-10	-30	-13
351.3	195.1	4.7	20-OH-LTB_4	-80	-10	-24	-15
375.3	153.1	5.4	22-OH-PD_1	-80	-10	-24	-15
375.3	221.1	5.4	22-OH-MaR_1	-80	-10	-24	-15
359.2	153.1	8.7	PD_1 / PDX $_1$	-80	-10	-21	-9
359.2	181.1	8.7	PD_1 / PDX $_2$	-80	-10	-19	-15
359.2	199.1	8.7	RvD_5	-80	-10	-21	-13
359.2	261.1	8.7	RvD_5 2	-80	-10	-20	-16
361.2	281.2	9.0	7,17-DiHDoPE (ω3) Lea 1	-110	-10	-22	-27
361.2	143.1	9.0	7,17-DiHDoPE (ω3) Lea 3	-110	-10	-23	-25
359.2	221.1	8.9	Maresin-1	-80	-10	-20	-16
359.2	250.1	8.9	MaR_1 2	-80	-10	-20	-16
359.2	101.1	9.3	RvD_6 / 4,14 1	-80	-10	-22	-16
359.2	159.1	9.3	RvD_6 / 4,14 2	-80	-10	-22	-16
333.3	199.1	7.5	RvE_2 / 5,15-diHEPE 1	-80	-10	-24	-17
333.3	253.1	7.5	RvE_2 / 5,15-diHEPE 2	-80	-10	-20	-12
333.3	201.2	8.7	RvE_3	-80	-10	-20	-12
333.3	245.1	8.7	RvE_3 2	-80	-10	-16	-14
335.2	195.1	8.7	LTB_4 Isomers	-80	-10	-22	-13
335.2	195.1	9.1	LTB_4	-80	-10	-22	-13

335.2	115.1	10.5	5*S*,6*R*-diHETE	-80	-10	-20	-13
335.3	235.1	8.7	5,15-diHETE 2	-80	-10	-22	-13
335.2	201.1	8.7	5,15-DiHETE Eico PAF	-50	-10	-30	-13
333.3	115.1	7.7	5,15-diHEPE	-80	-10	-22	-13
333.3	235.1	7.7	5,15-diHEPE 2	-80	-10	-22	-13
343.2	255.1	11.4	21-HDHA	-80	-10	-17	-14
343.2	245.1	11.5	17-HDHA	-80	-10	-17	-14
343.2	205.1	11.6	14-HDHA	-80	-10	-17	-14
343.2	193.1	11.5	13-HDHA	-80	-10	-17	-14
343.2	141.1	11.8	7-HDHA	-80	-10	-18	-15
343.2	101.1	12.3	4-HDHA	-80	-10	-17	-15
345.2	257.1	11.8	21-HDPA	-80	-10	-17	-14
345.2	247.1	11.9	17-HDPA	-80	-10	-17	-14
345.2	207.1	11.9	14-HDPA	-80	-10	-17	-14
345.2	195.1	11.9	13-HDPA	-80	-10	-17	-14
345.2	143.1	11.9	7-HDPA	-80	-10	-18	-15
317.2	259.1	10.4	18-HEPE	-80	-10	-16	-23
317.2	219.1	10.5	15-HEPE	-80	-10	-18	-12
317.2	179.1	10.7	12-HEPE	-80	-10	-19	-12
317.2	167.1	10.6	11-HEPE	-80	-10	-19	-12
317.2	115.1	11.0	5-HEPE	-80	-10	-18	-12
319.2	219.1	11.3	15-HETE	-80	-10	-19	-12
319.2	179.1	11.6	12-HETE	-80	-10	-21	-12
319.2	167.1	11.5	11-HETE	-80	-10	-21	-12
319.2	115.1	12.0	5-HETE	-80	-10	-21	-12
295.2	171.1	11.0	9-HODE	-60	-10	-19	-13
295.2	195.1	11.0	13-HODE	-60	-10	-25	-13
279.0	163.0	9.7	12-HHT	-30	-10	-30	-13
303.3	259.1	13.7	AA	-100	-10	-16	-18
301.3	257.1	13.5	EPA	-100	-10	-16	-18
327.3	283.1	13.7	DHA	-100	-10	-16	-18
329.3	285.1	13.8	DPA ω3 und ω6	-100	-10	-16	-18
335.2	113.2	7.9	PGB_1 I	-40	-5	-31	-11
335.2	221.2	7.9	PGB_1 II	-40	-5	-28	-11
349.0	113.0	5.6	15-keto-PGE_2	-80	-10	-25	-14
319.2	155.1	11.8	8-HETE	-50	-10	-18	-13
335.2	141.1	10.1	7,8-diHETE	-80	-10	-22	-13
335.2	109.1	9.5	5,8-diHETE	-80	-10	-21	-12
335.2	163.1	9.8	7,8-diHETE stereoisomer	-80	-10	-22	-13
337.2	163.1	10.5	7,8-diHETrE	-80	-10	-21	-12

SI-Table 6. Deuterated internal standard mix.

Q1	Q3	Retention Time [min]	Lipid Mediator [200 nM]	DP	EP	CE	CXP
327.3	116.1	12.0	d_8-5*S*-HETE	-80	-10	-17	-10
339.3	197.2	9.1	d_4-LTB_4	-80	-10	-22	-13
355.3	193.2	6.0	d_4-PGE_2	-80	-10	-25	-16
356.3	115.2	6.7	d_5-LXA_4	-80	-10	-19	-14
380.3	141.2	6.3	d_5-RvD_2	-80	-10	-23	-14
311.3	267.1	13.7	d_8-Arachidonic acid	-100	-10	-16	-18

SI-Table 7. Selection of external standards for quantification.

Q1	Q3	Retention Time [min]	Lipid Mediator	Calibration Curve [pg]	R^2	LOD [pg 10 µL^{-1}]
351.2	115.1	6.7	LXA_4	y=0.00005702x	0.9987	0.195
351.2	221.1	6.2	LXB_4	y=0.0001269x	0.9994	0.781
351.3	189.1	6.0	PGE_2	y=0.000101x	0.999	0.781
351.2	271.1	6.0	PGE_2 EicoPAF	y=0.0000436x	0.9991	0.781
351.3	233.1	6.2	PGD_2	y=0.00005004x	0.9993	0.781
353.3	193.1	6.4	$PGF_{2\alpha}$	y=0.000089x	0.9991	0.391
369.3	169.1	5.6	TXB_2	y=0.0000293x	0.9996	0.391
353.2	317.4	6.4	PGE_1 / PGD_1 I	Y=0.00002104x	0.999	0.195
353.2	273.4	6.4	PGE_1 / PGD_1 III	Y=0.00008255x	0.999	0195
351.3	195.1	4.7	20-OH-LTB_4	y=0.00005355x	0.9993	0.195
359.2	199.1	8.7	RvD_5	y=0.00008138x	0.9996	0.391
335.2	195.1	8.7	LTB_4 Isomers	y=0.00004057x	0.9992	0.781
335.2	195.1	9.1	LTB_4	y=0.00004202x	0.9998	0.781
335.2	115.1	10.5	5*S*,6*R*-diHETE	y=0.00005246x	0.999	0.781
335.3	235.1	8.7	5,15-diHETE 2	y=0.0004498x	0.9997	3.125
335.2	201.1	8.7	5,15-diHETE Eico PAF	y=0.0001274x	0.9991	1.563
317.2	115.1	11.0	5-HEPE	y=0.00004877x	0.9992	0.781
319.2	219.1	11.3	15-HETE	y=0.0001139x	0.9996	0.781
319.2	179.1	11.6	12-HETE	y=0.00004846x	0.9994	0.391
319.2	167.1	11.5	11-HETE	y=0.00001713x	0.9997	0.391
319.2	115.1	12.0	5-HETE	y=0.00004451x	0.9994	0.781
303.3	259.1	13.7	Arachidonic acid	y=0.0007155x	0.9993	1.563
301.3	257.1	13.5	EPA	y=0.0003602x	0.9996	1.563
327.3	283.1	13.7	DHA	y=0.0006402x	0.9502	3.125
329.3	285.1	13.8	DPA ω3 und ω6	y=0.0009207x	0.9996	1.563
319.2	155.1	11.8	8-HETE	y=0.00003942x	0.9996	1.563

SI-Table 8. Components PBS.

Name	Composure
Acidified water	Water, 230 mL<br>PBS-HCl, 2 mL
PBS-HCl	PBS (pH 7.4), 500 µL<br>HCl (1M), 30 µL
PBS (pH 7.4)	KH_2PO_4, 1.44 g L^{-1}<br>$Na_2HPO_4 \cdot 2H_2O$, 5.28 g L^{-1}<br>NaCl, 90 g L^{-1}

5. SI-References

[1] H. Jagusch, M. Werner, O. Werz, G. Pohnert, *Org. Lett.* **2019**, *21*, 4667-4670.

[2] O. Werz, J. Gerstmeier, S. Libreros, X. De la Rosa, M. Werner, P. C. Norris, N. Chiang, C. N. Serhan, *Nat. Commun.* **2018**, *9*, 59.

[3] J. Dalli, C. N. Serhan, *Blood* **2012**, *120*, e60-72.

[4] I. Vlasakov, P. Norris, J. Winkler, J. Dalli, C. Serhan, *Lipid Mediator Metabololipidomics LC-MS-MS spectra Book*, Brigham and Women’s Hospital and Harvard Medical School, Boston, **2016**.

6. NMR Spectra

- ^1H, ^{13}C, HSQC, ^1H,^1H-COSY, HMBC spectra for 15-hydroperoxy-PGE_2 **3**

^{1}H-NMR (600 MHz, *DMSO-d$_6$*) of 15-hydroperoxy-PGE$_2$ (**3**)

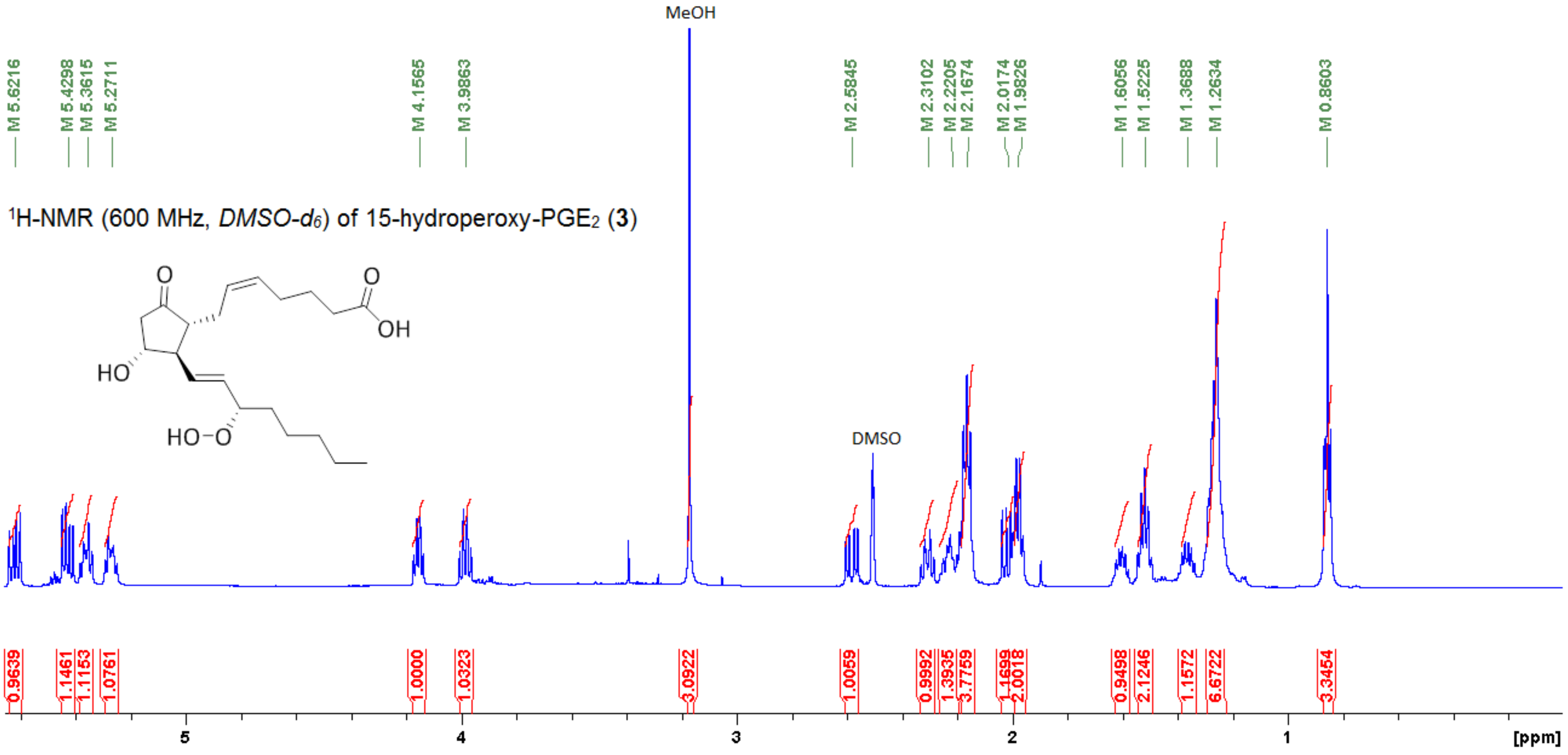

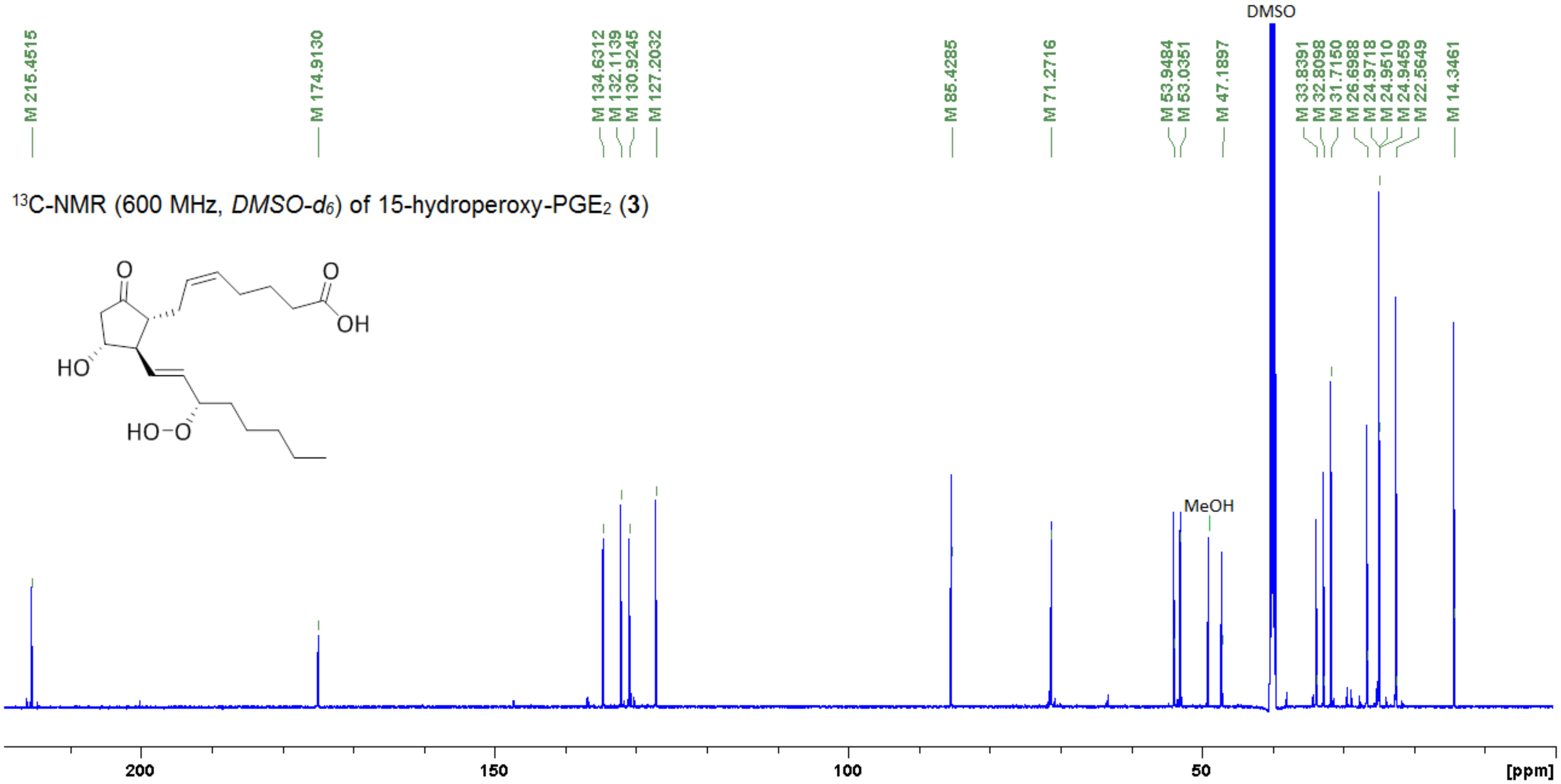
M 215.4515
M 174.9130
M 134.6312
M 132.1139
M 130.9245
M 127.2032
M 85.4285
M 71.2716
M 53.9484
M 53.0351
M 47.1897
DMSO
M 33.8391
M 32.8098
M 31.7150
M 26.6988
M 24.9718
M 24.9510
M 24.9459
M 22.5649
M 14.3461
^{13}C-NMR (600 MHz, *DMSO-d$_6$*) of 15-hydroperoxy-PGE$_2$ (**3**)
O
O
OH
HO
HO–O
MeOH
200
150
100
50
[ppm]

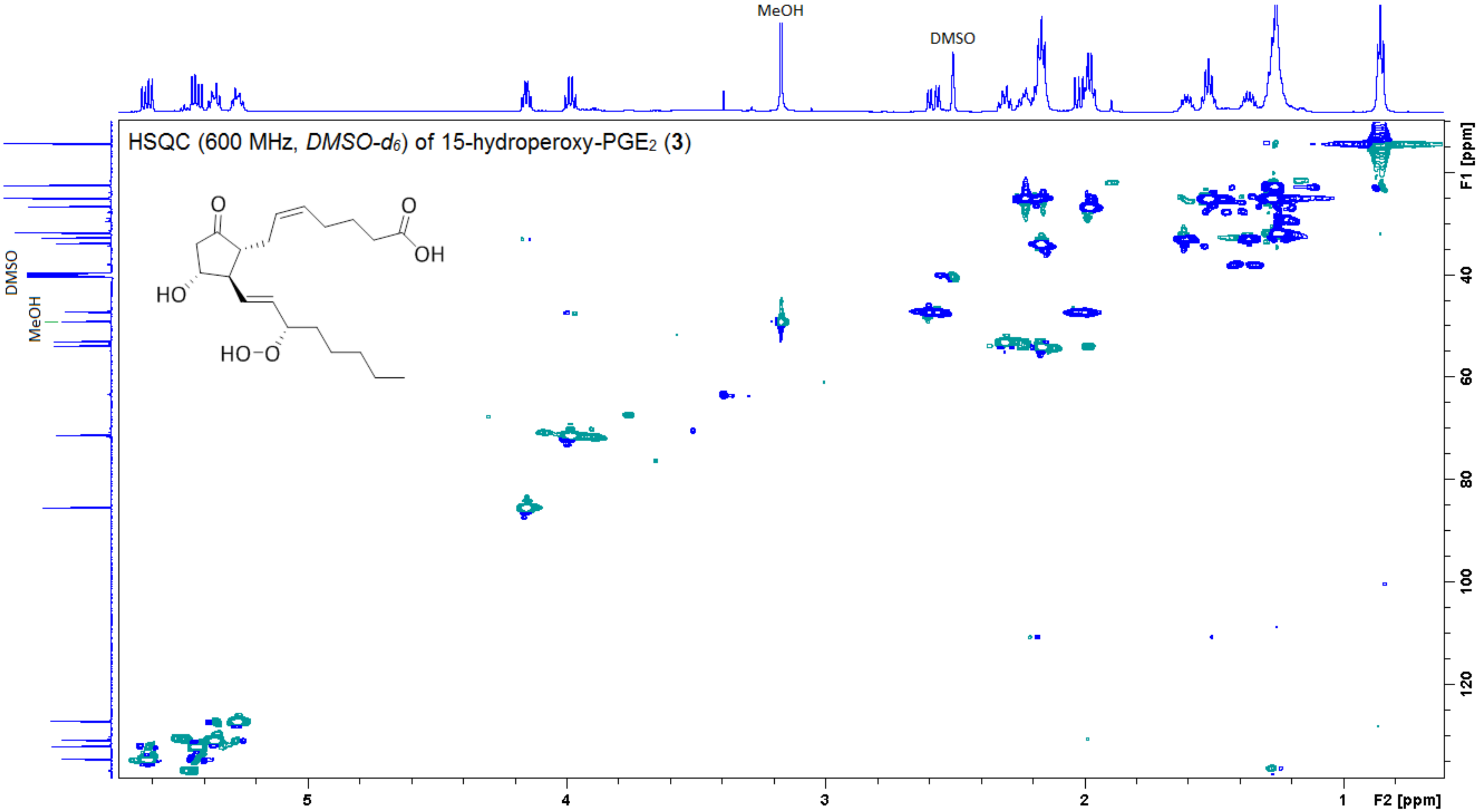

Note: the additional signals in the olefinic regions might result from degradation / isomerization during the extended measurement time.

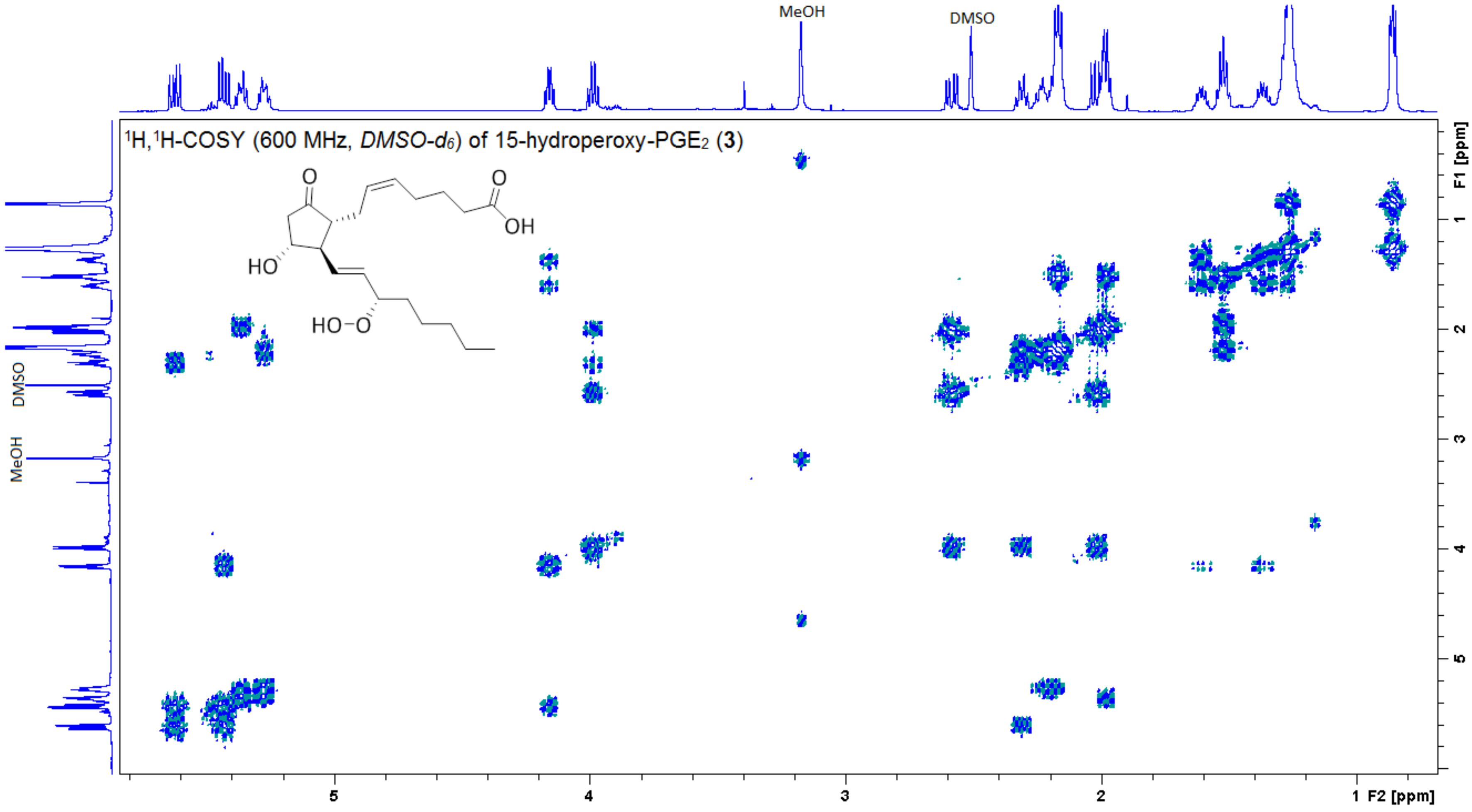

^{1}H,^{1}H-COSY (600 MHz, *DMSO-d6*) of 15-hydroperoxy-PGE2 (**3**)
MeOH
DMSO
O
O
OH
HO
HO−O
F1 [ppm]
F2 [ppm]

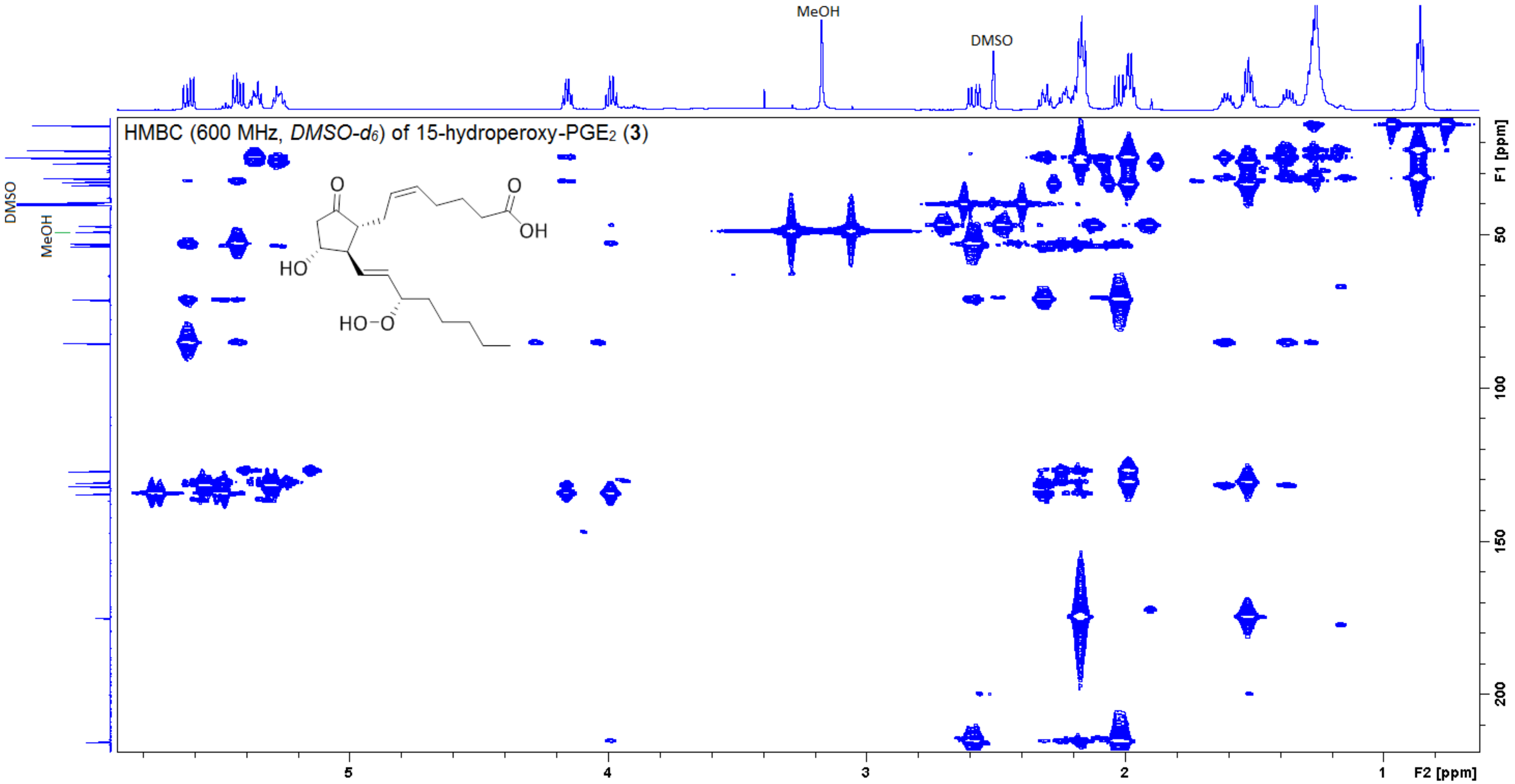

MeOH
DMSO
HMBC (600 MHz, DMSO-d6) of 15-hydroperoxy-PGE2 (3)
O
O
OH
HO
HO–O
F1 [ppm]
50
100
150
200
5
4
3
2
1
F2 [ppm]
